# Overcoming Batch Variability: A Chemically Defined XRS‐Based System for Ovine In Vitro Embryo Production

**DOI:** 10.1155/vmi/1718173

**Published:** 2026-06-05

**Authors:** Yun Wang, Enci Wang, Qingqing Chen, Gulibanu Nuermaimaiti, Anan Shi, Ye Xiao, Yuheng Luo, Yakun He, Wenhui Pi, Chunhe Xiang

**Affiliations:** ^1^ College of Animal Science and Technology, Shihezi University, Shihezi, 832003, China, shzu.edu.cn

**Keywords:** in vitro fertilization (IVF), in vitro maturation (IVM), ovine embryos, serum replacer, the 3Rs

## Abstract

Conventional IVM and IVF systems for sheep embryos rely on fetal bovine serum (FBS) and estrous sheep serum (ESS), which present challenges of batch variability, ethical concerns, and supply limitations. This study evaluated XRS Standard serum replacer as a complete substitute for both FBS and ESS in sheep IVEP. A 2 × 2 factorial design generated all four possible IVM/IVF combinations—FBS/ESS, FBS/XRS, XRS/ESS, and XRS/XRS (each additive at 10% v/v in IVM and IVF media). The FBS/ESS combination served as the conventional control. Cleavage rates (Day 2) and blastocyst development (Day 7) were quantified. XRS/XRS achieved 22.45% blastocyst rates—a 2.0‐fold improvement over controls (11.00%, *p* < 0.01). FBS/XRS showed intermediate efficacy (15.43%), whereas ESS‐supplemented groups exhibited reduced competence (12.71% and 11.00%, respectively). Total blastocyst cell numbers (122.1 ± 22.3 vs. 126.0 ± 22.4, *p* > 0.05) remained comparable across groups, demonstrating preserved embryo quality. This protocol eliminates animal‐derived components while maintaining developmental competence, aligning with the 3Rs (reduction, refinement, and replacement) and offering a scalable solution for commercial ovine embryo production.

## 1. Introduction

Ovine in vitro fertilization (IVF) is pivotal for accelerating genetic gain and reproductive efficiency [1, 2]. Conventional protocols supplement maturation medium with fetal bovine serum (FBS) promote oocyte maturation and fertilization medium with estrous sheep serum (ESS) to facilitate sperm capacitation [3–12]. However, reliance on animal‐derived sera introduces critical limitations: batch‐to‐batch variability, supply chain instability, and biosafety risks that hinder standardization and large‐scale application. For instance, ESS induces capacitation through cholesterol efflux and Ca^2+^ influx mechanisms, but high concentrations elevate apoptotic transcripts (e.g., BAX and caspase‐3) while impairing embryo quality [13, 14].

The inclusion of serum in embryo culture systems can no longer be considered acceptable due to these compounded drawbacks [15]. This paradigm necessitates the development of animal‐free alternatives that eliminate animal‐derived components while preserving developmental competence [16]. XRS Standard (XRSS10‐500, Xirui Infinity Biotechnology Co., Ltd., Hangzhou, China) represents a novel solution, engineered to minimize animal‐derived inputs and enhance batch consistency. Although FBS is conventionally used at 10% (v/v) for cell lines and primary cells, preliminary dose–response experiments identified 10% (v/v) XRS as optimal for supporting cellular proliferation and growth kinetics.

To the best of our knowledge, peer‐reviewed evaluations of XRS within mammalian in vitro embryo production systems remain limited. In an exploratory effort, this study examines the potential influence of XRS supplementation on cleavage rates and Day‐7 blastocyst development, with the modest aim of contributing toward a more reproducible, ethically sound, and scalable foundation for ovine embryo production.

## 2. Materials and Methods

### 2.1. Collection and Maturation of Oocytes

Ovaries were collected from a local abattoir from adult ewes of mixed meat‐type breeds slaughtered for human consumption; the animals were not selected by breed, genetic background, or coat type, and their exact breed composition and genetic characteristics were not recorded. Ovaries were transported to the laboratory within 4 h at 35°C in 0.9% NaCl. After three washes in warm saline, surface follicles (2–8 mm) were incised in 25‐mM HEPES‐buffered TCM‐199 (HEPES‐199, Wuhan Pricella Biotechnology Co., Ltd., China, PM150619) containing 2% FBS (FBS, BI, 04‐001‐1A/B/C), 2 U/mL heparin (Beijing Solarbio Science & Technology Co., Ltd., China, H8060), and 50 μg/mL gentamycin (40mg/mL, Yichang Humanwell Pharmaceutical Co., Ltd., China). Cumulus–oocyte complexes (COCs) displaying ≥ 2 intact cumulus layers and uniform cytoplasm were rinsed and allocated (80–100 per well) to 4‐well dishes with 0.6 mL pre‐equilibrated IVM medium: M199 supplemented with 10% (v/v) FBS or XRS Standard, 5 μg/mL follicle‐stimulating hormone (FSH; Jiangxi Haoran Bio‐Pharma Co., Ltd., China, 97048‐13‐0), 2 μg/mL LH (Ningbo Sansheng Biological Technology Co., Ltd., China, B160510), 1 mM sodium pyruvate (Gibco, 11360070), 2.0 mM Glutamax (Gibco, 35050061), and 40 μg/mL gentamicin, and then matured for 24 h in a humidified atmosphere at 38.5°C with 5% CO_2_.

### 2.2. IVF

Motile spermatozoa were isolated by swim‐up in a modified synthetic oviductal fluid (SOF) medium (107 mM NaCl, 7.17 mM KCl, 1.19 mM KH_2_PO_4_, 1.71 mM CaCl_2_, 0.49 mM MgCl_2_, 25.07 mM NaHCO_3_, 3.3 mM Na lactate, and 1.0 mM Na pyruvate; 270–280 mOsm kg^−1^, pH 7.2–7.3) [17] supplemented with 10% ESS or 10% XRS, 20 μg mL^−1^ heparin, 16 μM isoproterenol, and 50 μg mL^−1^ gentamicin. Cryopreserved semen (0.25 mL straws) was thawed at 37°C for 60 s; 200 μL aliquots were layered under 1.5 mL pre‐equilibrated SOF medium in 2‐mL tubes and incubated at 38.5°C, 5% CO_2_ for 50 min. The upper 1.4 mL containing motile cells was collected, centrifuged at 400 g for 6 min, resuspended in fresh medium to 5 × 10^6^ spermatozoa mL^−1^, and added to oocytes for 8 h under the same conditions. After fertilization, COCs were denuded by gentle pipetting in SOF‐HEPES, washed three times, and transferred to pre‐equilibrated in vitro culture (IVC) medium for continued culture.

Cell line statement: This study did not utilize any established cell lines. All experiments were performed using primary ovine COCs derived from ovaries obtained from a local abattoir and cryopreserved ram semen. The use of primary cells is essential for this research as it directly investigates the in vitro production of sheep embryos, reflecting physiological conditions relevant to ovine reproduction. The absence of cell lines does not impact the conclusions drawn regarding the efficacy of the XRS serum substitute in ovine in vitro embryo production (IVEP) systems.

### 2.3. IVC

Presumptive zygotes were pooled into groups of 60–80 and cultured in four‐well dishes containing 600 μL pre‐equilibrated modified SOFaai medium (270–280 mOsm kg^−1^, pH 7.2–7.3) overlaid with 400 μL mineral oil. The medium comprised SOF (107 mM NaCl, 7.17 mM KCl, 1.19 mM KH_2_PO_4_, 1.71 mM CaCl_2_, 0.49 mM MgCl_2_, 25.07 mM NaHCO_3_, 3.3 mM Na lactate, and 0.3 mM Na pyruvate) supplemented with 1 mM glutamine, 2.77 mM myo‐inositol, 1% (v/v) MEM amino acids (50×) (Gibco, 11130‐051), 1% (v/v) MEM nonessential amino acids (100×) (Gibco, 11140‐050), and 8 mg mL^−1^ fatty‐acid–free BSA (GPC Biotechnology, AA970‐25G). Cultures were maintained at 38.5°C in 5% CO_2_ with saturated humidity; developmental progression was assessed at 36 h (2‐cell) and on Day 7 (blastocyst).

### 2.4. Blastocyst Staining

For total cell counts, Day‐7 blastocysts were fixed with 4% paraformaldehyde at room temperature for 30 min, incubated in 10 μg mL^−1^ Hoechst 33342 (Sigma) for 30 min at room temperature, washed twice in PBS–0.1% BSA, mounted on slides, and immediately examined under a Leica DMi8 fluorescence microscope (excitation 330–380 nm) to quantify total nuclei.

### 2.5. Experimental Design

The study used a 2 × 2 factorial design in which M199 for IVM and SOF for IVF each received only one serum or alternative source at 10% (v/v), producing four exclusive combinations: FBS in IVM + ESS in IVF (control), FBS in IVM + XRS in IVF, XRS in IVM + ESS in IVF, and XRS in IVM + XRS in IVF, with five replicates of ≥ 50 COCs each to isolate and assess the effects of serum type at each stage while keeping the supplement concentration constant at 10% within its respective medium (Table 1).

**TABLE 1:** 2 × 2 factorial design for independent replacement of FBS and ESS by XRS during ovine IVM and IVF.

Groups	Factor		Treatment combination	Replicates (oocyte numbers)
	IVM	IVF		
FBS + ESS	FBS	ESS	FBS 10% + ESS 10%	5 (≥ 50 oocytes)
FBS + XRS	FBS	XRS	FBS 10% + XRS 10%	5 (≥ 50 oocytes)
XRS + ESS	XRS	ESS	XRS 10% + ESS 10%	5 (≥ 50 oocytes)
XRS + XRS	XRS	XRS	XRS 10% + XRS 10%	5 (≥ 50 oocytes)

### 2.6. Statistical Analysis

Statistical analyses were performed on IVEP‐derived data to evaluate the effects of replacing 10% (v/v) FBS and/or ESS with XRS during IVM and IVF. A two‐way ANOVA was used to test (i) the main effect of XRS inclusion and (ii) the interaction between XRS and serum type (FBS vs. ESS). Where significant main or interaction effects were detected (*p* ≤ 0.05), Tukey’s honestly significant difference (HSD) test was applied to identify specific pairwise differences among treatment groups. All analyses were conducted in SPSS (PASW Statistics v. 17.0, SPSS Inc., Chicago, IL, USA). Results are reported as means ± SEM unless stated otherwise.

## 3. Results

### 3.1. Comparable Full Cumulus Expansion After 24 h of In Vitro Maturation With FBS or XRS Standard

After 24 h of in vitro maturation in M199 medium supplemented with either 10% (v/v) FBS or XRS Standard, all COCs displayed complete cumulus expansion. No significant difference in the degree of cumulus expansion was observed between the two supplementation groups (Figure 1). The image of the mature oocyte was captured using a smartphone equipped with a stereomicroscope.

FIGURE 1Cumulus expansion of ovine oocytes matured for 24 h in vitro in (a) TCM199 with XRS; (b) TCM199 with FBS.

**Figure:**
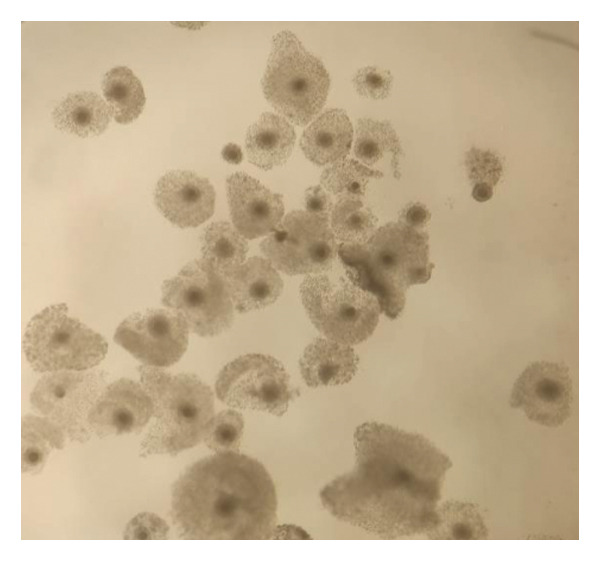
(a)

**Figure:**
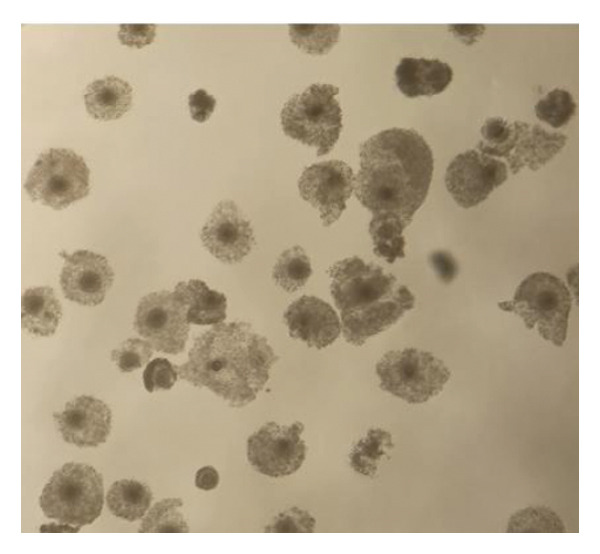
(b)

### 3.2. Impact of XRS Supplementation During IVM and IVF on Cleavage and Blastocyst Rate in Ovine Oocytes

**FIGURE 2:**
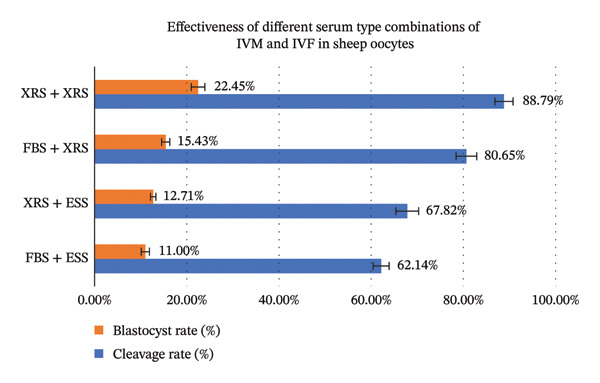
Developmental competence of ovine oocytes after IVM and IVF under defined combinations.

The impact of XRS on ovine IVEP was evaluated in a 2 × 2 factorial design that crossed XRS and conventional sera (FBS and ESS) during IVM and IVF. Developmental competence differed markedly among treatments (Table 2, Figure 2). The XRS–XRS regimen yielded the highest cleavage (88.79%) and blastocyst rates (22.45%, *p* < 0.05), followed by the FBS–XRS combination. In contrast, any protocol that included ESS—XRS–ESS or the traditional FBS–ESS—produced the lowest developmental indices; the FBS–ESS group was numerically the weakest, although differences were not statistically significant (*p* > 0.05).

**TABLE 2:** Cleavage and blastocyst rates (mean ± SEM) of ovine embryos after IVM–IVF under different XRS/serum combinations (*n* = 5 replicates).

Groups	Times	COCs (no.)	Cleaved embryos (no.)	Blastocysts (no.)	Cleavage rate (%)	Blastocyst rate (%)
FBS + ESS	5	54.80 ± 7.16	34.00 ± 4.06	6.00 ± 0.70	62.14 ± 1.73	11.00 ± 0.90
XRS + ESS	5	55.00 ± 6.82	37.20 ± 3.70	7.00 ± 1.00	67.82 ± 2.47	12.71 ± 0.59
FBS + XRS	5	54.60 ± 7.16	44.00 ± 5.57	8.40 ± 0.89	80.65 ± 2.24^a^	15.43 ± 0.88^b^
XRS + XRS	5	55.40 ± 7.63	49.20 ± 6.98	12.40 ± 1.52	88.79 ± 1.96^a^	22.45 ± 1.47^c^

*Note:* Superscripts denote differences relative to the conventional FBS–ESS control: ^a,b^ *p* ≤ 0.05; ^c^ *p* ≤ 0.01. Cleavage rate = cleaved embryos/COCs; blastocyst rate = blastocysts/COCs.

### 3.3. The Effects of Serum Replacer on Total Cell Number in Blastocysts

Total cell counts per blastocyst did not differ between the XRS and control groups (*p* > 0.05), indicating that XRS supplementation did not affect overall cellular proliferation during the preimplantation period (Table 3). Hoechst staining further confirmed that blastocyst nuclei were evenly distributed, supporting the conclusion that embryo quality was not impaired in the XRS group.

**TABLE 3:** Total cell number (mean ± SD) of ovine Day‐7 blastocysts produced under defined IVM–IVF serum regimens.

Groups	No. blastocysts	Blastocyst nuclei
FBS + ESS	6	122.17 ± 22.311
FBS + XRS	6	120.50 ± 17.964
XRS + ESS	8	126.00 ± 22.418
XRS + XRS	10	127.40 ± 19.438

## 4. Discussion

### 4.1. XRS Serum Substitute Outperforms Conventional Sera in Ovine IVEP

COCs displayed complete cumulus expansion in M199 medium supplemented with 10% (v/v) XRS Standard. Comprehensive substitution of FBS in IVM and ESS in IVF with XRS (XRS–XRS) significantly improved all key developmental parameters of ovine IVEP, increasing cleavage to 88.79% and blastocyst yield to 22.45% (*p* ≤ 0.01 vs. FBS–ESS). XRS is a commercially available, standardized serum substitute; the manufacturer has disclosed that it includes albumin, transferrin, a defined lipid mixture, a full complement of amino acids, vitamins, electrolytes, carbohydrates, and fatty acids, but no quantitative data were provided. These reported components are intended to collectively support oocyte maturation and embryo development in vitro. This improvement suggests that the standardized formulation of XRS may more closely align with the metabolic and signaling requirements of ovine oocytes than the variable protein milieu present in conventional sera.

Replacing only the IVF medium (FBS–XRS) produced intermediate outcomes (80.65% cleavage; 15.43% blastocyst), suggesting that the benefit of XRS is cumulative: Optimization during IVM is necessary but not sufficient; removal of ESS during IVF provides an additional gain. Conversely, retention of ESS consistently depressed development (≤ 67.82% cleavage; ≤ 11.00% blastocyst), regardless of whether it was paired with FBS or XRS. This negative effect aligns with transcriptomic evidence that 10% ESS upregulates proapoptotic and oxidative‐stress pathways in ovine blastocysts [12]. Elevated reactive oxygen species during IVF may therefore compromise subsequent embryo integrity when ESS is present.

### 4.2. XRS Boosts Ovine Blastocyst Output Without Diminishing Per‐Embryo Cell Count

Beyond developmental kinetics, total blastocyst cell number was unaffected by XRS (*p* > 0.05), demonstrating that the higher blastocyst yield was achieved without compromising individual embryo quality. The present findings complement parallel work in bovine IVF where another serum replacer enhanced blastocyst development capacity and cryotolerance [18]. Extending such comparative studies across species will clarify whether XRS offers universal advantages for livestock IVEP or whether species‐specific refinement of its formulation is required.

### 4.3. XRS Enables Safe, Standardized, and Animal‐Free Ovine IVEP Aligned With One Health

Equally important, the use of a chemically defined substitute abolishes variation inherent to serum, improving protocol reproducibility—a critical requirement for commercial programs and for compliance with 3Rs principles [19]. The high seroprevalence of zoonotic abortifacient pathogens—*Brucella* (70.7%), *Leptospira* spp. (55.2%), and *Chlamydia abortus* (21.9%)—in sheep populations underscores livestock systems as critical reservoirs for human–animal disease transmission, particularly in resource‐limited settings where protective practices are inadequate (e.g., 80% of farmers report lacking gloves or masks) [20]. Traditional IVEP relies on animal‐derived sera such as FBS or ESS, which pose biosafety risks and exhibit inherent biological variability. The adoption of XRS serum replacer presents a transformative alternative that aligns with One Health principles by mitigating zoonotic exposure and enhancing standardization.

Substituting both FBS and ESS with XRS delivers a superior, more consistent, and safer ovine IVEP system. Future investigations should focus on the molecular mechanisms—e.g., growth‐factor signaling and redox homeostasis—through which XRS exerts its beneficial effects, and on optimizing concentration and timing to further elevate embryo quality and cryotolerance. Such insights will underpin the transition of ovine IVEP toward fully defined, animal‐free, and globally portable protocols.

### 4.4. ESS Toxicity and Oocyte Quality, Not XRS, Limit Ovine Blastocyst Output

Low blastocyst formation remains a significant bottleneck in ovine IVEP. Our data demonstrate that this inefficiency is critically linked to the inclusion of ESS in the fertilization medium. While ESS is conventionally used to support sperm capacitation, its presence exerts a pronounced negative impact on embryonic development. In our system, residual ESS consistently restricted cleavage rates to ≤ 67.82% and blastocyst yields to ≤ 12.71%, irrespective of oocyte source or morphological selection rigor. This detrimental effect is particularly exacerbated when oocytes are collected outside the optimal breeding season or lack stringent morphological grading.

The underlying impairment appears to be driven by ESS‐triggered cytotoxicity, chiefly via oxidative‐stress generation and the ensuing activation of proapoptotic cascades. Previous work has shown that supplementing sheep IVF media with ESS markedly remodels blastocyst quality at the molecular level, downregulating antioxidant transcripts while upregulating apoptotic markers [12]. In that study, embryos exposed to 10% ESS reached a blastocyst rate of roughly 20.8%, and those exposed to 2% ESS reached ∼15.9% [12]. In our hands, however, blastocyst yields never exceeded 11%. In contrast, cleavage rates showed no significant difference (≤ 62.14% in our study vs. 63.8% reported under 12.71% ESS conditions), suggesting that ESS exerts its primary inhibitory effects during the postcleavage phase. The substantial reduction in blastocyst development (Table 2) strongly indicates enhanced susceptibility of our IVEP system to ESS‐induced suppression, potentially exacerbated by inherent variability in oocyte quality derived from slaughterhouse‐derived ovaries and less stringent selection criteria during COC retrieval. The observed discrepancy in ESS sensitivity compared to previous studies may be attributed to variations in oocyte quality parameters, particularly mitochondrial integrity and cortical granule distribution, which have been shown to modulate embryo developmental competence.

Whether ESS is paired with FBS or only partially replaced by chemically defined supplements (XRS), the persistence of serum components proves inhibitory. However, a pivotal solution emerges: the complete substitution of both FBS and ESS with a fully defined XRS system. This approach doubled our blastocyst yield to 22.45%, effectively overcoming the serum‐imposed barrier. Importantly, this increase was achieved without compromising embryo quality, as evidenced by statistically equivalent total cell counts and inner cell mass to trophectoderm (ICM:TE) ratios compared to serum‐containing controls.

Therefore, the inefficiency observed in traditional ovine IVEP stems not from inherent limitations of defined culture systems but rather from the cytotoxic effects of ESS, amplified by nonideal oocyte procurement. Eliminating serum entirely via XRS represents a viable strategy to significantly enhance blastocyst production efficiency while maintaining developmental competence.

## 5. Conclusions

The 2 × 2 factorial experiment demonstrates that complete replacement of FBS in IVM and ESS in IVF with XRS (XRS–XRS) significantly elevates cleavage and blastocyst rates without compromising total cell number. This defined, serum‐free regimen therefore provides a robust, reproducible, and biosafe platform for ovine IVEP and establishes a transferable reference for optimizing embryo production protocols in other livestock species.

## Author Contributions

Y.W., E.W., G.N., A.S., and Y.X. conducted the experiments and analyzed the data; Q.C., Y.L., and Y.H. assisted with part of the experimental work; W.P. conceived the study, designed and performed the experiments, analyzed the data, and wrote the manuscript; C.X. acquired funding and providing cryopreserved semen.

## Funding

This research was funded by the Startup Foundation for Bingtuan Science and Technology Project (2025AA015, NYHXGG2023AA101) and the Science and Technology Specialist Team Service Program of Shihezi University (KJTP202401).

## Disclosure

All authors read and approved the final manuscript.

## Ethics Statement

All experimental procedures were performed in strict accordance with the European Directive 2010/63/EU and were approved by the Biology Ethics Committee of Shihezi University, Xinjiang, China (approval no. A2024‐302).

## Conflicts of Interest

The authors declare no conflicts of interest.

## Data Availability

All data are included in this article.
